# An experimental study of the acute effects of visual and olfactory nature stimuli on task performance

**DOI:** 10.3389/fpsyg.2026.1872507

**Published:** 2026-07-13

**Authors:** Sarayu Chandra Mouli, Marco Palma, Suma Katabattuni, Jay E. Maddock

**Affiliations:** 1 Department of Environmental and Occupational Health, Center for Health and Nature, Texas A&M School of Public Health, Texas A&M University, College Station, TX, United States; 2 Human Behavior Laboratory, Texas A&M Department of Agricultural Economics, Texas A&M University, College Station, TX, United States; 3 Department of Computer Science, Texas A&M University, College Station, TX, United States

**Keywords:** behavioral economics, cognition, experimental psychology, nature contact, performance, stress

## Abstract

**Introduction:**

Nature exposure has been associated with improvements in physiological, emotional, and cognitive functioning. However, workplace research has primarily focused on visual access to nature, despite nature being inherently a multisensory experience. Drawing on Attention Restoration Theory (ART), Stress Reduction Theory (SRT), and emerging work on olfactory environments (“smellscapes”), this study examines how visual and olfactory nature stimuli independently and jointly influence cognitive and behavioral outcomes in workplace settings.

**Methods:**

A between-subjects experiment (*N* = 256) employed a 2 × 2 design (visual: present vs. absent × olfactory: present vs. absent). Participants completed tasks assessing attention, memory recall, abstract reasoning, risk aversion, and dishonest behavior. Outcomes were analyzed using two-way factorial ANOVA to evaluate the independent and combined effects of exposures, with supplementary one-way ANOVA conducted across the four conditions. Blink rate was also recorded via continuous eye-tracking as a physiological indicator of cognitive load, alongside facial expression data to contextualize affective responses.

**Results:**

Effects varied by sensory condition and outcome. Visual exposure was associated with improvements in attention and abstract reasoning, whereas olfactory exposure was associated with memory performance and dishonest responding. Significant interactions were observed for several outcomes, including attention, memory, risk-taking, and dishonest responding. Blink rates were generally lower under nature exposure conditions than in the control condition. Physiological measures indicated reduced blink rates under nature exposure conditions relative to control environments, suggesting differences in visual attention and task engagement across experimental conditions.

**Discussion:**

These findings suggest that multisensory nature exposure differentially influences cognitive and behavioral processes in workplace environments rather than producing uniform benefits. By demonstrating that low-cost, passive interventions such as visual and olfactory cues can shape attention, decision-making, and mental overload, this study advances environmental psychology and supports the integration of multisensory design strategies to enhance workplace functioning and overall productivity.

## 1 Introduction

People spend 90% of their time inside buildings (Evans and McCoy, 1998), making indoor environments a dominant determinant in shaping human health, cognition and behavior. The benefits of nature contact have been well documented in existing literature. Research in various fields including environmental psychology, architectural design, and public health consistently emphasize that exposure to natural environments improves mental health, reduces stress, enhances cognitive function, and promotes physical health. While the influence of the built environment on physical and mental health has gained increasing attention, its role in shaping everyday cognitive and behavioral functioning particularly in workplace settings remains less clearly understood (Kellert and Wilson, 1995; Salingaros, 2015).

This is particularly relevant with modern life increasingly isolating people from nature. With more time being spent indoors at home or in work environments (Jimenez et al., 2021), this disconnection, especially in office settings, has been linked to reduced wellbeing, heightened stress, lower job satisfaction, and decreased productivity among employees (Jimenez et al., 2021; Pluut and Wonders, 2020). Literature linking nature and health have been conducted through various meta and systematic analysis and have yielded numerous studies in the last two decades (Coventry et al., 2021; De Croon et al., 2005; Heerwagen, 2009; Patwary et al., 2024; Yao et al., 2021a; Yao et al., 2021b). Foundational effects of nature on health were initially conducted within healthcare settings to show that hospital patients staying in patient rooms with a view of nature had shorter recovery times and required fewer painkillers (Andrade and Devlin, 2015; Ulrich et al., 1991; Ulrich, 1984). More recent work has extended these findings to workplace contexts, where access to greenery, natural light, and indoor plants has been linked to improved cognitive performance and mental wellbeing.

Terrapin Bright Green, a sustainability consultancy firm reported a 15% productivity increase over 3 months (cognitive tests, self-reporting, work tasks completed, etc.) for employees with access to natural elements, compared to those without (Ryan et al., 2014). Additionally, indoor plants have been shown to reduce carbon dioxide and dioxin levels by 10% in air-conditioned spaces and 25% in naturally ventilated buildings (Tarran et al., 2007). Conversely, the absence of natural elements, particularly greenery, has been linked to reduced wellbeing and increased discord among employees (Velazquez, 2019). Despite this substantial evidence, offices consistently overlook the importance of nature indirectly impacting employee mental health and productivity, resulting in economic loss. Some attribute this gap to limited resources, and planning abilities, often highlighting how much of the existing literature emphasizes resource-intensive interventions. This leaves a gap in understanding how low-cost, passive nature-based strategies can be applied in real-world workplace settings. Building on this limitation, the present study focuses on performance outcomes in environments that reflect contemporary, resource-constrained office contexts.

Building on these broader workplace implications, prior research has also examined how nature exposure influences specific cognitive and behavioral outcomes. Studies focused on cognitive processes including attention and memory spans along with tendency to cheat and take precautionary risks. Three studies reported that exposure to nature can be invigorating for attention and both short- and long-term memory span (Hartig et al., 2003; Marc et al., 2008; Pilotti et al., 2015). Herzog’s study also mentions the combination of attentional capacity and increased cognition which would bring down anti-social behaviors like cheating while Berman and colleagues found that exposure to nature can enhance self-control, which may reduce tendencies to engage in cheating behavior, a phenomenon relevant in the workplace (Berman et al., 2012).

**FIGURE 1:**
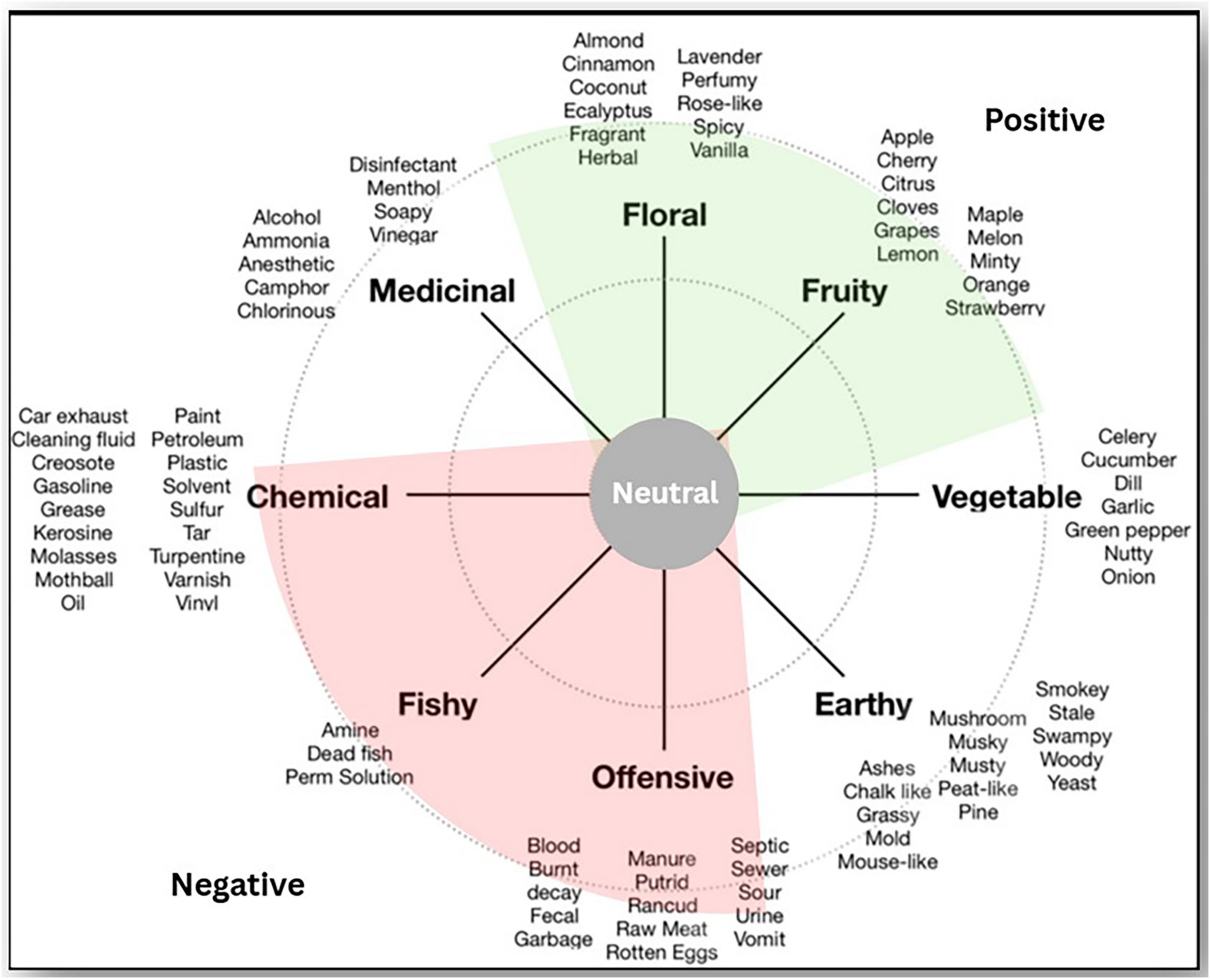
Scent spectrum: positive scents vs. negative scents. Adapted from Lindborg and Liew (2021), this figure illustrates the spectrum of olfactory’ stimuli ranging from pleasant, nature-associated scents (e.g., floral and fruity) to unpleasant or nuisance-related odors (e.g., chemical, fishy, and offensive). The highlighted regions represent the positive (green) and negative (red) domains of olfactory-perception.

Beyond visual elements, scents from nature also significantly represent an important but comparatively understudied pathway through which nature may influence wellbeing and cognitive performance. Recent theoretical frameworks, such as the one presented by Marselle and colleagues in 2021, suggests that specific sensory traits, such as scent, may influence multiple dimensions of wellbeing through distinct pathways, including cognitive restoration (Bentley et al., 2023). For example, essential oils from plants like lavender and eucalyptus, commonly used in aromatherapy promote relaxation and reduce anxiety (Amakura et al., 2002; Masuo et al., 2021; Toet et al., 2010). Within workplace settings, aromatic scents could boost productivity and improve mental clarity. The first study correlating nature scents to health outcomes (1995) examined how odor-evoked memories can influence attention and emotional responses, shaping cognitive and affective processes (Herz and Cupchik, 1995; Tennessen and Cimprich, 1995). Recent work found that certain nature-related scents, such as the smell of rain (geosmin) or the fragrance of trees like pine and cedar, can trigger powerful positive memories, emotional responses, stress reduction and cognitive functions (Herz, 2016). Olfactory environments span a wide spectrum ranging from pleasant, nature-associated scents to unpleasant or nuisance-related odors, each eliciting distinct cognitive and affective responses (Lindborg and Liew, 2021). Figure 1 illustrates this distribution of olfactory stimuli and highlights the positioning of nature-based scents within positive sensory domains. The influence of multisensory nature stimuli strengthens the case for nature-based design strategies, as improvements in cognitive functioning and emotional wellbeing may lead to greater employee engagement, reduced stress, and enhanced workplace efficiency.

## 2 Purpose and research questions

The present study investigates how visual and olfactory nature stimuli independently and in combination affect workplace-relevant cognitive processes and decision-making behaviors. Using a 2 × 2 experimental design (*visual exposure: present vs. absent × olfactory exposure: present vs. absent*), participants were randomly assigned to one of four conditions as below (Table 1).

**TABLE 1:** Experimental design and treatment conditions.

Stimuli	No exposure to nature views	Exposure to nature views
No scents	Group 1 (Control): Complete absence of nature (closed curtains)	Group 2: Nature Views through Windows
Nature Scent	Group 3: No views, w/scent	Group 4: Nature View w/scent (Both present).

Participants were randomly assigned to one of four conditions in a 2 × 2 design varying visual exposure (nature views vs. no views) and olfactory exposure (nature scent vs. no scent).

To capture distinct dimensions of workplace functioning, outcomes were organized into domains:

1. cognitive processes, including attention, memory performance, and abstract reasoning; and
2. decision-making behaviors, including risk aversion and dishonest behavior.

Based on prior literature on nature exposure and multisensory environmental effects, the following hypotheses were tested:

Cognitive domain:

*H1a*: Exposure to visual nature stimuli will improve cognitive performance (attention, memory, and reasoning) relative to no exposure.

*H1b*: Exposure to olfactory nature stimuli will improve cognitive performance relative to no exposure.

*H1c*: Combined visual and olfactory exposure will produce greater improvements in cognitive performance than either condition alone.

Behavioral domain:

*H2a*: Exposure to visual nature stimuli will be associated with reduced dishonest responding and altered risk aversion relative to no exposure.

*H2b*: Exposure to olfactory nature stimuli will be associated with reduced dishonest responding and altered risk aversion relative to no exposure.

*H2c*: Combined exposure will produce stronger behavioral effects than either condition alone.

### 2.1 Physiological evaluation

In addition to behavioral outcomes, physiological measures were collected to provide objective indicators of attentional engagement, cognitive processing, and affective responses. Eye-tracking and facial expression data were recorded using iMotions, a neurophysiological platform widely used to assess gaze behavior and emotional responses in behavioral research (Farnsworth, 2020; Palma, 2022).

#### 2.1.1 Blink rate (BR) analysis

Blink rate (BR) was included as a physiological measure associated with visual attention, cognitive processing, and task engagement. Prior research has reported relationships between blink rate and cognitive demand; however, findings remain highly context dependent, with both increased and decreased blink rates observed under different experimental conditions (Fogarty and Stern, 1989; Karson, 1983). In visually demanding cognitive tasks, lower blink rates have been associated with sustained visual attention and uninterrupted information processing, as blinking temporarily interrupts visual input (Franěk and Petružálek, 2025; Joye and van den Berg, 2018). Accordingly, blink-rate findings in the present study are interpreted as a context-specific indicator of attentional engagement rather than a direct measure of cognitive load or mental effort.

#### 2.1.2 Facial expressions analysis

Facial expressions were analyzed to identify a range of emotional expressions, including positive emotions (e.g., joy, engagement, smiles) and negative emotions (e.g., anger, sadness, contempt), providing insight into participants’ affective responses during task performance. The following hypotheses were tested for facial behaviors:

*H3*: Participants exposed to nature stimuli (visual, olfactory, or combined) will exhibit lower blink rates compared to the control condition, reflecting differences in attentional engagement and visual processing during task performance.

*H4* (Exploratory): To examine whether exposure to visual and olfactory nature stimuli is associated with differences in facial-expression patterns measured using AFFDEX emotion recognition software.

## 3 Materials and methods

### 3.1 Study design overview

This study employed a 2 × 2 between-subjects experimental design to examine the independent and combined effects of visual and olfactory nature stimuli on cognitive, behavioral, and physiological outcomes. Across all conditions, participants completed a structured sequence of six incentivized tasks. Each of these tasks were meant to assess measures of cognitive performance like attention, memory and reasoning along with physiological responses like facial expressions. Experimental sessions were conducted in small groups (8–12 participants), with each session assigned to a single condition to ensure consistent exposure throughout the study. To support cognitive reset and recovery, short, standardized breaks were incorporated between tasks while maintaining the assigned environmental condition.

### 3.2 Sample and setting

The study was conducted in a laboratory located adjacent to a large green space called the “Research Park.” The setting had views of this area on all sides, allowing controlled access to natural views. Participants were undergraduate students ( ≥ 18 years) recruited through the internal SONA system. Eligibility criteria included normal or corrected-to-normal vision. Exclusion criteria included conditions incompatible with eye-tracking such as photosensitive epilepsy or pacemakers. Participants who did not adhere to study protocols including the use of external aids (e.g., mobile phones), incomplete task responses, or interaction with other participants were excluded from the final analysis to minimize potential confounding effects.

**FIGURE 2:**
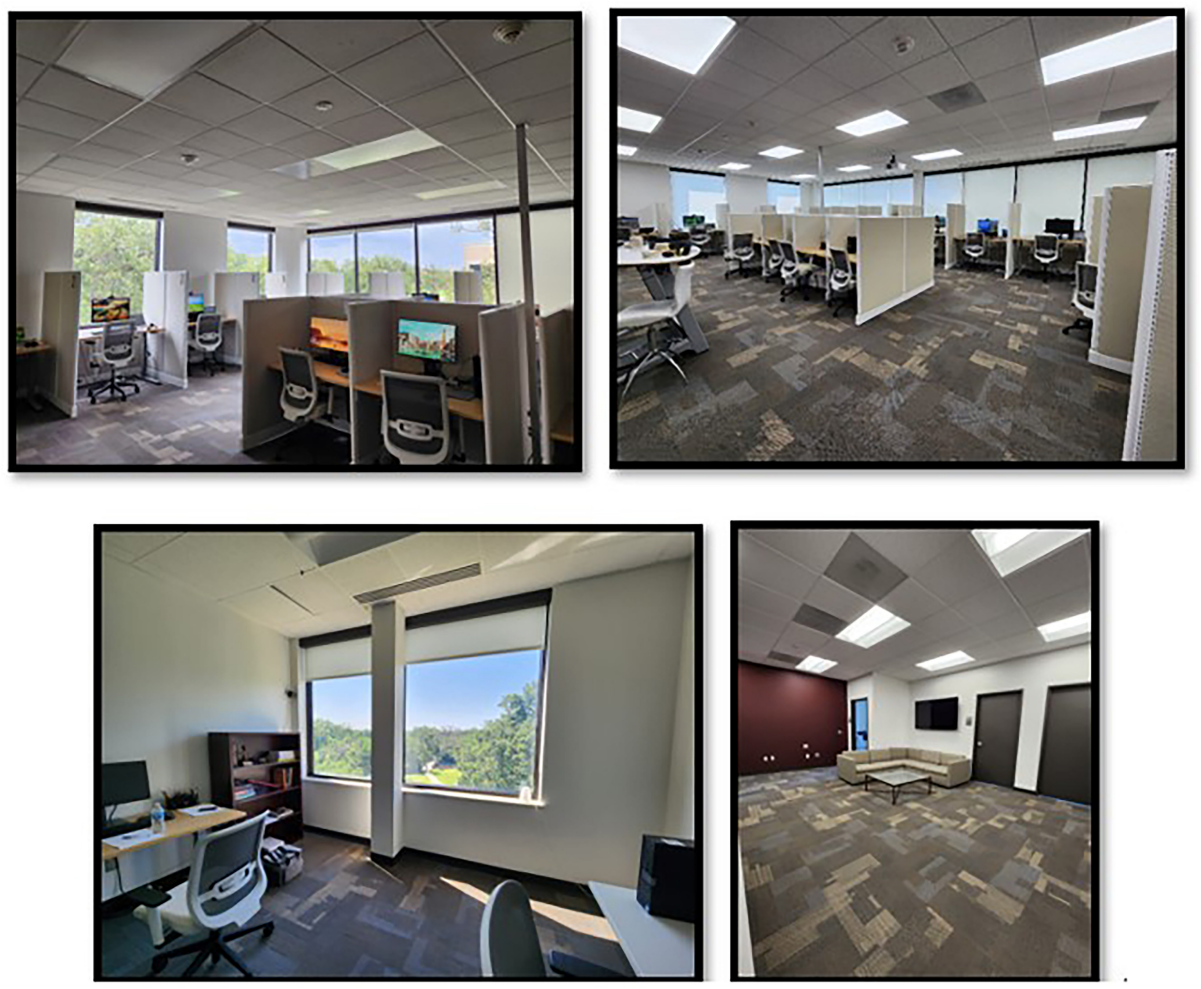
Study settings condition-wise (from left to right: top left: views session, top right: control, bottom left: views/both break room, bottom right: control/scents break room). Representative images of the lab environments used in the study. These settings illustrate how environmental exposure was systematically manipulated across conditions during both task performance and inter-task breaks. Top left: views condition with open blinds providing visual access to the surrounding natural environment. Top right: control condition with blinds closed, limiting exposure to external views. Bottom left: break room used for the views and combined (views + scent) conditions, with access to natural views. Bottom right: break room used for the control and scent-only conditions, without visual access to nature.

Participants were randomly assigned to one of four experimental conditions. In the control condition, participants completed the tasks with blinds closed and no olfactory stimuli. The breaks were also given in a windowless breakroom within the laboratory. In the visual condition, blinds were opened to provide wide views of the surrounding green space for both task performance and break periods. In the olfactory condition, a “scent-wave” diffuser was used to deliver a nature-based scent via a cold-air diffusion system while windows remained blocked. Diffusers were placed in the break rooms with the windows closed (Figure 2).

### 3.3 Experimental procedure

The experimental tasks were implemented using O-Tree, an open-source Python-based platform for behavioral experiments (Chen et al., 2016). Physiological data, including eye-tracking and facial expressions, were recorded using iMotions 9.3, which integrates multiple data streams in real time through a local host system. Participants completed the study in sessions, each assigned to a single experimental condition. Within each session, participants worked independently at private computer terminals. At the beginning of each session, eye-tracking calibration was performed using a standard 9-point calibration procedure to ensure accurate physiological measurements.

**FIGURE 3:**
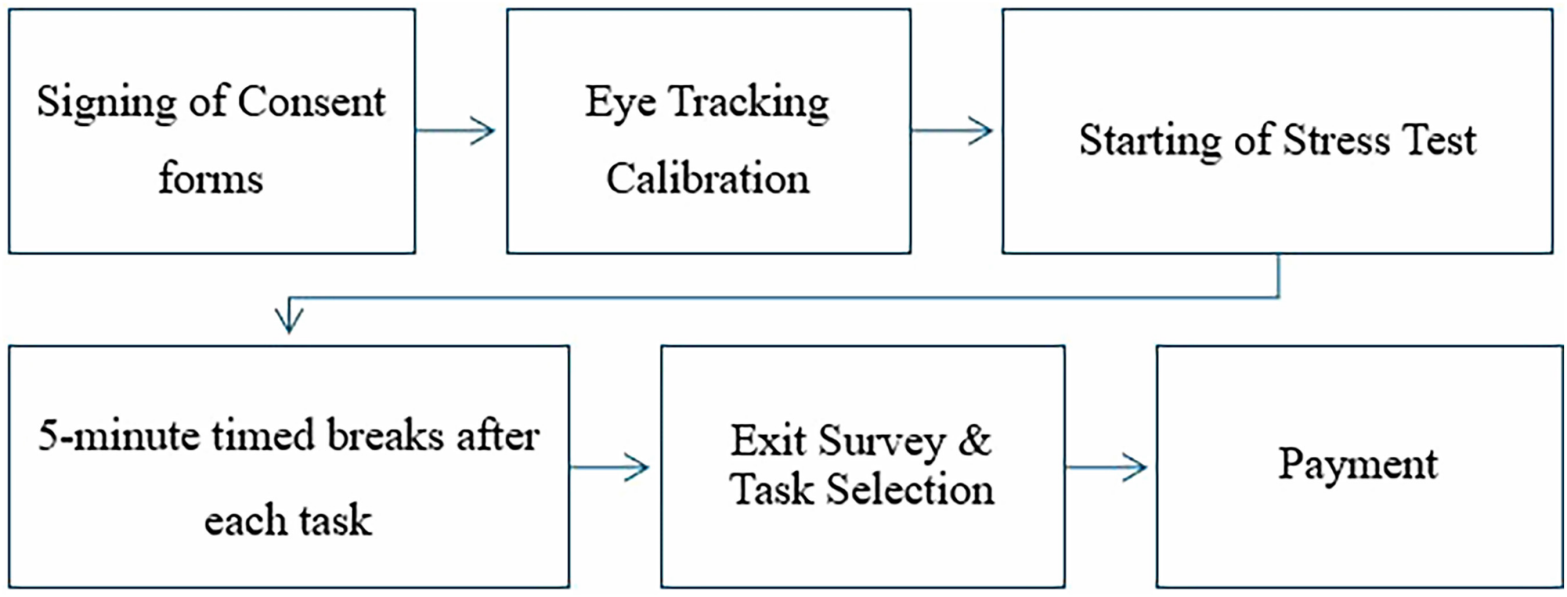
Procedural flow of a typical session. Overview of the sequence of procedures followed during each experimental session. Participants first completed informed consent, followed by eye-tracking calibration. All participants then began the stress test simultaneously, after which they completed a series of task-based measures in a fixed order. Short, standardized 5-minute breaks were provided between tasks. At the conclusion of the session, participants completed an exit survey, followed by random task selection for incentive-based payment.

Participants completed six task-based measures presented in a fixed sequence. The session began with a stress-inducing task, followed by tasks assessing attention, memory recall, abstract reasoning, risk aversion, and honesty. This sequence was intentionally standardized across all participants to control baseline stress levels and to minimize variability arising from task order or fatigue effects. By maintaining consistent progression, all participants experienced the same cognitive trajectory throughout the session, allowing for more reliable comparisons across experimental conditions. Between tasks, participants were provided with standardized 5-min breaks in a secondary breakroom. These breaks were designed to allow for brief cognitive reset and recovery, while also maintaining exposure to the assigned environmental condition (visual, olfactory, combined, or control), as described previously. Figure 3 illustrates the procedural flow of a typical session.

In the olfactory condition, a scent diffuser was used to introduce a nature-based fragrance into the experimental environment. The device employed cold-air diffusion technology, which breaks down essential and fragrance oils into fine particles that are dispersed into the air through a fan and nozzle system. This allowed for an even distribution of scent within the space at approximately 2 ppm, ensuring subtle exposure without overwhelming the indoor environment, as higher concentrations may lead to discomfort (e.g., headaches or irritation) and potentially affect task performance. The commercially available fragrance “Walk in the Woods” was selected to evoke sensory characteristics commonly associated with natural outdoor environments. The fragrance contained top notes of Calabrian bergamot and base notes of amber, woods, and musk. While designed to evoke associations with forest settings, the fragrance does not represent a direct replication of naturally occurring environmental odors and should therefore be interpreted as a nature-associated olfactory stimulus rather than authentic nature exposure. In the combined condition, both the scent diffuser and visual exposure (open blinds) were provided.

All procedures were conducted in accordance with the ethical standards of the Institutional Review Board and the Declaration of Helsinki and its subsequent amendments. The study protocol was approved by the University’s Institutional Review Board prior to participant recruitment.

An a priori power analysis indicated that a sample size of 64 participants per condition would be sufficient to detect a medium effect size (Cohen’s *d* ≈ 0.5) with 80% power at an α level of 0.05. This estimate is consistent with prior experimental studies examining the effects of nature exposure on affective and cognitive outcomes, which have reported effect sizes in the range of d ∼0.40–0.60 (Gaekwad et al., 2022; Li et al., 2022). The final sample of 256 participants therefore provided adequate power to detect meaningful differences across conditions.

### 3.4 Incentive framework

Participants were informed that, although they would complete all six tasks, only one task would be randomly selected for monetary compensation. At the end of each session, a six-sided die was rolled to determine the incentivized task, and participants were compensated based on their performance in that task. To ensure consistency in motivation across tasks, potential earnings were calibrated to be comparable across all six tasks. Participants received base compensation of $15 for participation, with an additional performance-based bonus of up to $12. Thus, all participants had the opportunity to earn up to $27, regardless of which task was selected. This incentive structure was designed to maintain equal engagement across tasks while minimizing bias toward any single task due to differences in expected rewards. Performance on tasks was quantified using standardized metrics, including accuracy, response time, and task completion measures. In addition, physiological data (eye-tracking and facial expression analysis) were collected to provide complementary indicators of cognitive load and affective responses during task performance.

## 4 Outcome measures

The study assessed both behavioral and physiological outcomes to capture task performance and underlying cognitive and affective processes across conditions. Behavioral measures were derived from participants’ performance on the six tasks, while physiological measures were obtained using eye-tracking and facial expression analysis.

### 4.1 Behavioral measures

Each task was extracted from the literature to measure a specific key domain relating to workplace functioning. Cognitive performance was measured through attention retention, memory recall, and abstract reasoning. Decision-making behaviors were tested through risk aversion and cheating. Quantitative performance metrics based on accuracy, response time, and task completion.

#### 4.1.1 Task 1: mental math (stress test)

The first task was used to induce cognitive stress and assess performance under pressure, based on an adapted version of the Trier Social Stress Test (Ferreira, 2019; Gunnar et al., 2021; Meier et al., 2022). Participants completed a timed arithmetic task involving sequential subtraction of a two-digit number from a four-digit number across multiple rounds. Each response was time-limited, requiring rapid and continuous calculation. Task 1 served primarily as a stress-induction procedure intended to elicit cognitive and physiological stress prior to the subsequent experimental tasks. Although performance on the task was recorded and analyzed, its primary purpose was to create a standardized stressor rather than function as a standalone assessment of workplace-related cognitive performance. Performance was measured based on accuracy and number of correct responses completed under time constraints. Incentives were performance-based, with correct answers contributing to token earnings.

#### 4.1.2 Task 2: letter identification (attention skills)

This task assessed attentional control. Participants were required to count the number of occurrences of a target letter within short text passages presented on screen, a validated task to measure short-term attention retention (Zhang et al., 2019). The task spanned approximately 10 min. Performance was measured using accuracy and response time, capturing both correctness and processing speed.

#### 4.1.3 Task 3: word list learning (memory skills and recall)

This task measured short-term memory and recall ability, adapted from the Wechsler Adult Intelligence Scale (WAIS) subtest (Axelrod, 2001), measured memory and recall efficiency. Participants viewed a 15-word list selected from a set of 50–60 common words from the repeatable episodic memory test (Parker et al., 1995) for 15 s. They were then given 45 s to recall as many words as possible repeating this process over four rounds. They could recall up to 15 words per round, with excess entries triggering a system reset. Performance was quantified based on the number of correctly recalled words across repeated trials. For payoff, one round was selected, with each correct word earning one token ($0.75), totaling up to $12 with perfect recall.

#### 4.1.4 Task 4: Raven’s test (abstract reasoning and intelligence)

Adapted from the WAIS subtest and Raven’s Progressive Matrices (Axelrod, 2001), our version was condensed to a 7-min test, including a 1-min practice session. The task featured 3× 3 matrices, with participants completing pattern-recognition problems of increasing difficulty, selecting the correct option to complete each matrix. Performance was evaluated based on accuracy and the number of problems successfully completed. Scoring included rounds completed and accuracy, with each correct response earning a token ($0.50), up to a maximum of $10, weighted for advanced levels. The purpose of this task was to assess abstract reasoning and fluid intelligence which play a vital role in problem solving and innovation.

#### 4.1.5 Task 5: Holt and Laury risk assessment (risk aversion)

Risk preferences were assessed using Holt and Laury (2002) lottery choice task (Holt and Laury, 2002), a widely used experimental measure of risk aversion. Participants completed 10 sequential decisions between a safer option (Option A, e.g., 10% chance of $2, 90% chance of $1.60) and a riskier alternative (Option B, e.g., 10% chance of $3.85, 90% chance of $0.10), with probabilities of higher payoffs increasing across rounds. Risk aversion was operationalized as the switching point at which participants shifted from the safer to the riskier option. Earlier switching indicates greater risk aversion, while later switching reflects greater risk tolerance. To ensure incentive compatibility, one decision was randomly selected for payment at the end of the session using a 10-sided die roll, and earnings were based on the outcome of the selected choice.

#### 4.1.6 Task 6: cheating task

Cheating was assessed using a reporting task that allowed participants to misreport outcomes for personal gain. Participants were told they were paired with another participant (Player 2), although all interactions were with a computer-simulated partner to ensure consistency. In each round, participants (Player 1) were endowed with 10 tokens and shown a randomly generated number (0–10) indicating the amount to be shared. While instructed to report this value, participants could report a different number, thereby retaining a larger share. Cheating was measured as the deviation between assigned and reported values across rounds.

The task consisted of 20+ rounds, with one round randomly selected for payment to maintain incentive compatibility. A probabilistic extension (20% chance of additional rounds) was incorporated (Oechssler and Sofianos, 2019). This approach provides a continuous measure of dishonest behavior and is widely used in experimental research on economic decision-making (Dai et al., 2018).

### 4.2 Physiological measures

The Tobii X2-60 eye-tracking device was attached to each monitor used in the experiment. Using infrared lights to reflect patterns in participants’ corneas, it measured total fixation duration and gaze time, operating at 60 measurements per second. Blink rate (BR) was included as a physiological measure associated with visual attention, cognitive processing, and task engagement. The analysis incorporated cognitive, visual, and memory tasks, with data segmented by condition and task for each participant. Variations in blink rate were examined across tasks and experimental conditions to assess differences in attentional engagement during task performance. The literature surrounding blink-rate interpretation is mixed, with some studies reporting lower blink rates under conditions of increased cognitive demand and others reporting the opposite. Across this literature, the most consistent conclusion is that blink-rate findings are highly context dependent and should be interpreted within the specific visual and cognitive demands of the task being examined.

Additionally, the study incorporated AFFDEX, a tool within iMotions, to analyze facial expressions and gain a better understanding of participants’ emotional reactions during the eye-tracking sessions. Facial expressions are indicators of participants’ affective responses during task performance. Expression data were derived from facial movements, including changes in the eyebrows, eyes, cheeks, and mouth, using automated tracking of facial landmarks. The analysis was based on the Facial Action Coding System (FACS), developed by Ekman and Friesen in 1971 (revised in the 1980s) which decomposes facial expressions into discrete Action Units (AUs) (Boehm-Davis et al., 2000; Ekman and Friesen, 1978; Ekman et al., 1987; Ekman, 1999; Paul et al., 1983). Each AU corresponds to a specific facial muscle movement (e.g., eyebrow raise, lip movement; Supplementary Table 1), allowing for systematic identification of observable expressions. The AFFDEX algorithm maps these AUs to emotional indicators using the EMFACS framework (Ekman and Friesen, 1978; Ekman, 1999; Kjærstad et al., 2020). While facial expressions do not directly measure internal emotional states, prior research suggests that they provide reliable, real-time indicators of observable affective responses and engagement during cognitive tasks (De Gelder, 2006; Vaessen et al., 2019). Thus, facial expression data were used to assess relative differences in positive and negative effects across experimental conditions (Karson, 1983; Stöckli et al., 2018). The iMotions platform processes raw data related to these emotions and levels of engagement, using a 10% threshold for accuracy (Kjærstad et al., 2020).Together, eye-tracking and facial expression measures provided complementary physiological indicators of cognitive and affective responses under different environmental conditions. Facial expression measures were treated as exploratory outcomes and were summarized descriptively to provide supplementary insight into participants’ affective responses across experimental conditions.

## 5 Data analysis

Behavioral outcomes were primarily analyzed using two-way factorial ANOVA to evaluate the independent effects of visual exposure, olfactory exposure, and their interaction. One-way ANOVA and Tukey HSD comparisons across the four experimental conditions were conducted as supplementary analyses to facilitate interpretation of condition-specific differences. All statistical analyses were conducted using IBM SPSS Statistics (V.29). Formally, the two-way factorial ANOVA model was expressed as:


Y=β⁢0+β⁢1⁢(V⁢i⁢e⁢w⁢s)+β⁢2⁢(S⁢c⁢e⁢n⁢t)+β⁢3⁢(V⁢i⁢e⁢w⁢s×S⁢c⁢e⁢n⁢t)+ε,

where Y represents the outcome measure, β_0_ is the intercept, β_1_ and β_2_ represent the main effects of visual and olfactory exposure, β_3_ represents the interaction effect, and ε is the error term. For comparison across the four experimental conditions, one-way ANOVA was additionally conducted using the model:


Y⁢i⁢j=μ+α⁢i+ε⁢i⁢j,

where *Y*_*ij*_represents the outcome for participant *j*in condition *i*, μis the overall mean, α_*i*_represents the effect of condition *i*, and ε_*ij*_is the error term. For statistically significant omnibus results (*p* < 0.05), post hoc pairwise comparisons were performed using Tukey’s Honestly Significant Difference (HSD) test to identify specific differences between conditions. Effect sizes (η^2^) and 95% confidence intervals were reported where appropriate.

For behavioral tasks, performance metrics include accuracy, response time, and task-specific scoring measures, depending on the nature of each task. Risk aversion was operationalized as the switching point in the Holt and Laury task, and dishonest behavior was quantified as the deviation between assigned and reported values in the cheating task.

Physiological data were analyzed to examine variations in cognitive load and affective responses across conditions. Blink rate was aggregated at the task level for each participant and compared across conditions to assess differences in cognitive demand. Facial expression data were summarized as relative indicators of affective responses (e.g., attention, engagement, and positive expressions) and examined descriptively across experimental conditions. All statistical tests were evaluated at a significance level of α = 0.05.

## 6 Results

Results are presented across the four experimental conditions, beginning with participant characteristics, followed by task-based performance and physiological outcomes. Descriptive statistics for all outcome variables across conditions are presented in Table 2. Overall, descriptive patterns indicate that performance on cognitive tasks (attention, memory, and reasoning) tend to be higher in the nature-exposed conditions compared to the control group, while stress (Task 1) and risk aversion (Task 5) show minimal variation across conditions. Cheating (Task 6) appears lower in the scent and combined conditions relative to control. Supplementary Table 2 summarizes the demographic characteristics of the 256 participants across the four experimental conditions. The sample was predominantly under 25 years of age (80%), with a near-equal distribution by sex. In terms of race/ethnicity, 40% of participants identified as Asian and 23% as White. Approximately 46% were undergraduate students, while nearly half (∼50%) were pursuing graduate-level education.

**TABLE 2:** Descriptive statistics of task performance across experimental conditions.

Outcome	Control (Mean ± SD)	Views (Mean ± SD)	Scents (Mean ± SD)	Both (Mean ± SD)
Stress (Task 1)	1.06 ± 0.39	1.03 ± 0.18	1.03 ± 0.18	1.02 ± 0.13
Attention (Task 2)	1.89 ± 0.44	2.27 ± 0.57	2.03 ± 0.50	2.09 ± 0.53
Memory (Task 3)	2.51 ± 0.97	3.39 ± 1.07	3.56 ± 1.05	3.06 ± 1.09
Reasoning (Task 4)	2.94 ± 1.30	3.34 ± 1.54	3.34 ± 1.38	3.62 ± 1.11
Risk aversion (Task 5)	1.11 ± 0.32	1.02 ± 0.13	1.03 ± 0.18	1.11 ± 0.32
Cheating (Task 6)	2.55 ± 1.60	2.14 ± 1.53	1.56 ± 0.96	1.95 ± 1.28

Values are presented as mean ± standard deviation for each task across the four experimental conditions. Higher scores indicate better performance for cognitive tasks. Stress, attention, and abstract reasoning outcomes were based on predefined performance categories, while memory performance reflects aggregate recall scores.

Most participants reported adequate baseline conditions, with over 90% indicating a good night’s sleep prior to the session and 56% reporting a relaxed state before participation. Following the stress-inducing task (mental math), 52% of participants reported increased perceived stress levels. No statistically significant differences were observed in demographic or baseline characteristics across the four conditions, suggesting successful randomization.

### 6.1 Task performance analysis

**FIGURE 4:**
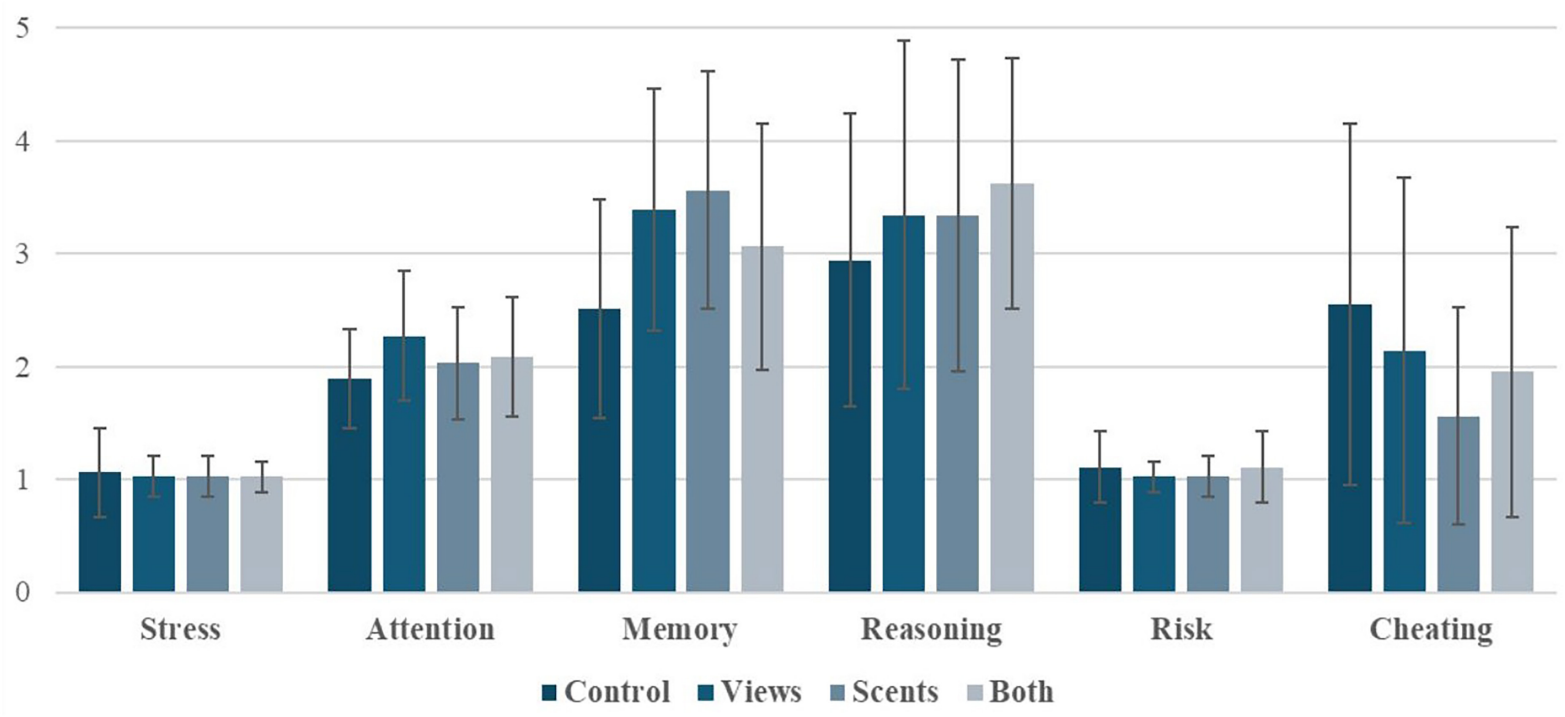
Condition-wise task performance across experimental groups. Bar chart illustrating mean performances across the six tasks, for each experimental condition. Nature-exposed conditions generally show a better I higher performance in cognitive tasks and lower cheating compared to the control group.

Results from the two-way factorial ANOVA are presented in Table 3. Significant main effects of visual exposure were observed for attention retention and abstract reasoning, whereas significant olfactory effects were observed for memory recall and dishonest responding. Significant visual × olfactory interactions were observed for attention retention, memory recall, precautionary risk-taking, and dishonest responding, indicating that the effects of one sensory modality depended on the presence of the other. No significant main effects or interactions were observed for the stress-inducing task. Supplementary mixed-effects models incorporating session-level random intercepts produced findings that were largely consistent with the primary factorial analyses, suggesting minimal influence of session-level clustering on study conclusions (Supplementary Table 6). To facilitate interpretation of condition-specific differences, one-way ANOVAs and Tukey *post-hoc* comparisons were also conducted across the four experimental groups, with Tukey’s HSD *post-hoc* comparisons reported (Supplementary Tables 3, 4). Mean scores were comparable across groups for both stress (Mcontrol = 1.06; Mviews = 1.03; Mscents = 1.03; Mboth = 1.02) and risk aversion (Mcontrol = 1.11; Mviews = 1.02; Mscents = 1.03; Mboth = 1.11). Significant differences were observed for attention (Task 2), memory (Task 3), abstract reasoning (Task 4), and honesty (Task 6). For attention (Task 2), participants in the views condition (M = 2.27) showed higher performance compared to the control group (M = 1.89), with additional differences observed relative to the scents (*M* = 2.03) and combined conditions (*M* = 2.09). For memory (Task 3), all nature-exposed conditions showed higher recall performance compared to the control group (*M* = 2.51), with the views condition (*M* = 3.39) exhibiting the highest mean performance, followed by the scents (*M* = 3.56) and combined conditions (*M* = 3.06). For abstract reasoning (Task 4), the combined condition (*M* = 3.63) showed higher performance compared to the control group (*M* = 2.94), as well as relative to both the views (*M* = 3.34) and scents (*M* = 3.34) conditions. In Task 6 (cheating), the scents condition (*M* = 1.56) showed the greatest reduction in dishonest behavior, with 38.8% less cheating compared to control (*M* = 2.55), while the combined condition (*M* = 1.95) showed a 23.5% reduction compared to control. These patterns are visually summarized in Figure 4, which illustrates condition-wise differences in mean task performance across all six tasks, highlighting improved cognitive outcomes in nature-exposed conditions relative to control.

**TABLE 3:** Two-way factorial ANOVA results.

Outcome	Effect	*F*(df1, df2)	*p*-value	Partial η^2^
Task 1: stress test	Views	*F*(1, 252) = 0.61	0.437	0.002
	Scents	*F*(1, 252) = 0.61	0.437	0.002
	Views × Scents	*F*(1, 252) = 0.07	0.795	0.000
Task 2: attention retention	Views	*F*(1, 252) = 11.68	<0.001	0.044
	Scents	*F*(1, 252) = 0.06	0.807	0.000
	Views × Scents	*F*(1, 252) = 5.96	0.015	0.023
Task 3: memory recall	Views	*F*(1, 252) = 2.03	0.155	0.008
	Scents	*F*(1, 252) = 7.47	0.007	0.029
Task 4: abstract reasoning	Views × Scents	*F*(1, 252) = 27.35	<0.001	0.098
	Views	*F*(1, 252) = 4.22	0.041	0.016
	Scents	*F*(1, 252) = 4.22	0.041	0.016
	Views × Scents	*F*(1, 252) = 0.14	0.709	0.001
Task 5: precautionary risk-taking	Views	*F*(1, 252) = 0.06	0.801	0.000
	Scents	*F*(1, 252) = 0.06	0.801	0.000
	Views × Scents	*F*(1, 252) = 7.74	0.006	0.030
Task 6: dishonest responding	Views	*F*(1, 251) = 0.00	0.969	0.000
	Scents	*F*(1, 251) = 11.78	< 0.001	0.045
	Views × Scents	*F*(1, 251) = 5.40	0.021	0.021

Views = visual nature exposure; Scents = olfactory nature exposure. F statistics are reported with numerator and denominator degrees of freedom. Factorial analyses were conducted to evaluate the independent and combined effects of visual nature exposure (views), olfactory nature exposure (scents), and their interaction across cognitive and behavioral outcomes. Effect sizes are reported as partial eta-squared (η^2^).

## 7 Blink rate analysis

**FIGURE 5:**
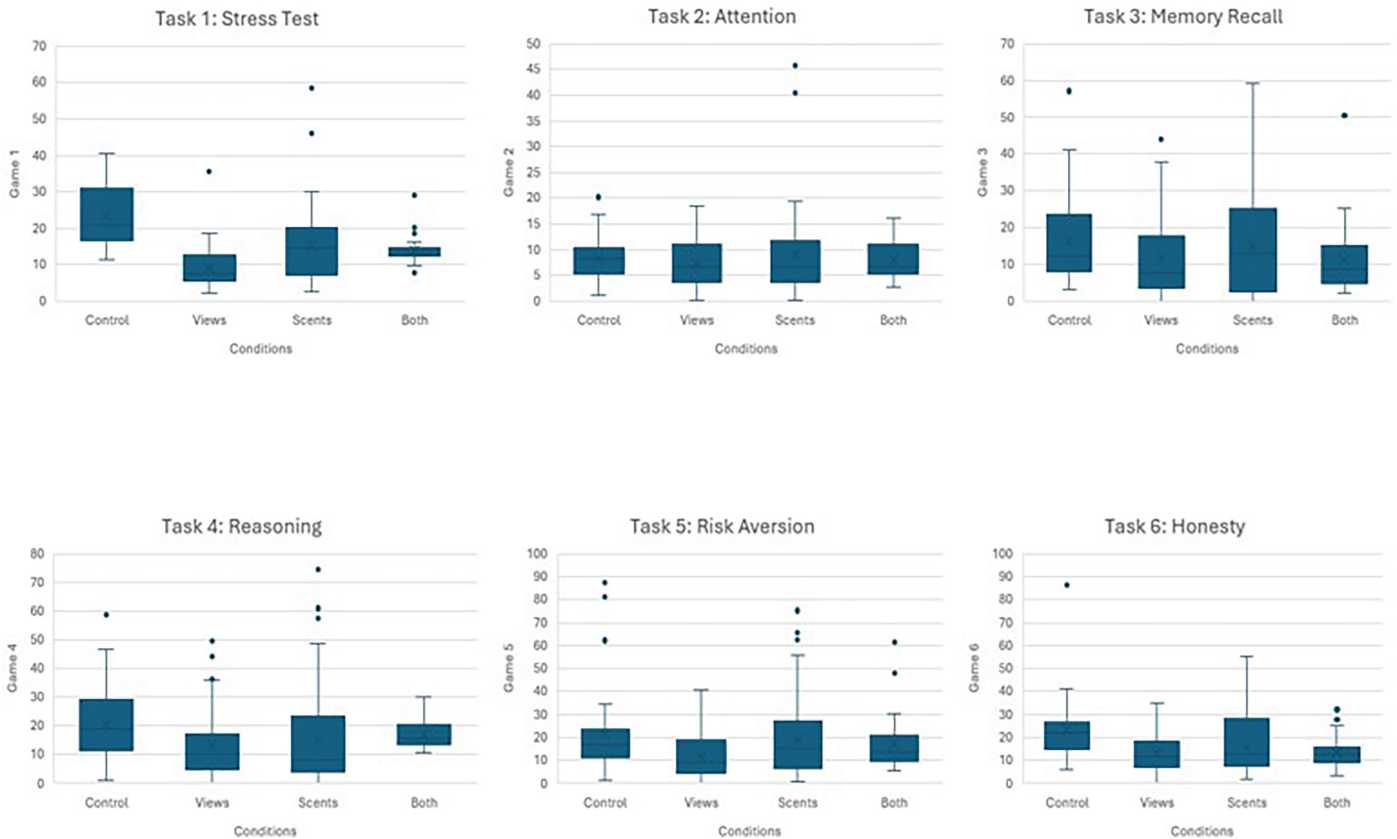
Blink rate differences per task across condition. Boxplots illustrating the distribution of performance scores for each of the six tasks across the four experimental conditions (control, views, scents, and combined). Boxes represent the interquartile range (IQR), horizontal lines indicate median values, and “×” markers denote mean values. Whiskers extend to the minimum and maximum values within 1.5× IQR, with points representing outliers. These plots provide a visual comparison of central tendency and variability in performance across conditions.

Blink rate (BR) data showed substantial variability across tasks and conditions, consistent with prior research reporting average blink rates of approximately 12–15 blinks per minute (Tsubota and Nakamori, 1993). Across four of the six tasks, participants in the nature exposure conditions exhibited lower blink rates compared to the control group. In visually demanding cognitive tasks, lower blink rates have been associated with sustained visual attention and uninterrupted information processing, although blink-rate interpretation remains highly context dependent. While blink-rate findings were used primarily as a complementary physiological measure, observed differences were supported by small-to-moderate effect sizes (η^2^ = 0.04–0.28) (Figure 5 and Table 4). Because the experimental tasks required sustained visual attention to on-screen stimuli, blink-rate patterns in the present study are interpreted primarily within the context of attentional engagement and visual information processing rather than as a direct measure of cognitive load alone. Collectively, these findings suggest that exposure to nature was associated with differences in attentional engagement and physiological responses.

**TABLE 4:** Blink Rates per condition with significance and effect sizes.

Condition	Task 1	Task 2	Task 3	Task 4	Task 5	Task 6
	(*p* < 0.01)			(*p* = 0.02)	(*p* < 0.001)	(*p* < 0.001)
	η^2^ = 0.281			η^2^ = 0.043	η^2^ = 0.072	η^2^ = 0.12
Control	23.46	8.25	16.37	20.40	21.56	23.27
Views	14.36	7.70	13.49	16.03	15.38	17.07
Scents	13.07	8.88	14.91	14.88	18.91	15.78
Both	13.84	8.01	11.19	17.05	17.07	13.53

Mean blink rate values across six tasks for each condition. Statistically significant differences and corresponding effect sizes (η^2^) are indicated where applicable.

Facial expression data were additionally aggregated to examine the relative presence of key affective responses, including attention, engagement, sentimentality, joy, and surprise (Table 5). These outputs were examined descriptively to provide supplementary physiological context and were not analyzed as primary inferential outcomes. Across nature-exposed conditions (3 conditions out of 4), higher levels of attention and joy were observed, with engagement more prominent in the views and combined conditions, and sentimentality highest in the scents condition. Negative emotional responses (e.g., anger, fear, disgust) showed minimal variation across conditions and are therefore provided as Supplementary Table 5.

**TABLE 5:** Global presence (%) of emotional responses.

Emotional responses	Attention	Engagement	Sentimentality	Joy	Smile	Surprise
Control	29.9	7.1	2.4	0.60	1.0	0.9
Views	43.8	29.0	2.6	7.3	7.0	4.5
Scents	41.1	12.2	13.8	8.5	5.1	6.6
Combined	48.3	25.8	2.3	5.8	5.6	4.9

Percentage presence of detected emotional responses across experimental conditions, derived from AFFDEX analysis. Values represent aggregated proportions of each response category across all tasks.

## 8 Discussion

In this study, visual and olfactory nature stimuli were associated with differential effects on cognitive and behavioral outcomes in simulated workplace tasks. Participants exposed to nature stimuli demonstrated improvements in attention, memory recall, and abstract reasoning, along with lower levels of dishonest behavior. These findings suggest that nature-based environmental cues may influence both cognitive performance and decision-making processes in task-based settings.

These findings are consistent with established frameworks such as Attention Restoration Theory (ART) (Kaplan, 1995), which emphasizes the role of natural environments in replenishing directed attentional resources, and Stress Reduction Theory (SRT) (Ulrich et al., 1991), which highlights the restorative effects of nature exposure on psychological and physiological functioning. The observed improvements in attention, memory, and abstract reasoning are particularly consistent with ART, as these tasks rely heavily on directed cognitive resources that may benefit from restoration following exposure to natural stimuli. The stronger cognitive effects observed under visual nature exposure are also aligned with ART, which has traditionally emphasized visual engagement with natural environments as a mechanism supporting attentional recovery. SRT provides a complementary explanation for the physiological findings. Although performance on the stress-inducing task did not differ significantly across conditions, the physiological patterns observed across nature exposure groups suggest that nature-based stimuli may influence underlying affective and attentional processes even when behavioral differences are not immediately detectable. While these findings should be interpreted cautiously, they are broadly consistent with the restorative mechanisms proposed by SRT.

The present findings also contribute to a growing body of literature suggesting that restorative responses are not limited to visual engagement with nature. Recent research has demonstrated that multiple sensory modalities, including visual, auditory, and olfactory stimuli, can independently contribute to restorative experiences within indoor environments. Yildirim et al. (2024) reported modality-specific restorative effects across sensory channels in workplace settings, highlighting the importance of considering restoration as a multisensory process rather than a purely visual phenomenon (Yildirim et al., 2024). Similarly, Sakimura et al. (2025) demonstrated physiological restoration in the absence of visual nature elements, challenging traditional vision-centric interpretations of restorative theory (Sakimura et al., 2025). These findings are further supported by theoretical work suggesting that restoration may occur through mechanisms extending beyond attentional recovery alone and may involve broader perceptual, affective, and associative processes (Joye and Dewitte, 2018). Collectively, this emerging literature provides important context for the modality-specific effects observed in the present study and supports a broader multisensory understanding of restorative environments. At the same time, the effects observed in this study were not uniform across outcomes. Rather than producing consistent improvements across all measures, nature exposure generated task-specific benefits, suggesting that different sensory modalities may influence distinct cognitive, behavioral, and physiological processes. Furthermore, certain findings, particularly the reductions in dishonest behavior associated with olfactory exposure, may involve mechanisms that extend beyond traditional restoration theories. However, because the present study did not directly assess moral cognition, implicit associations, affective mediation, or related psychological constructs, the specific processes underlying these behavioral effects remain unclear and warrant further investigation.

Two findings stand out. First, factorial analyses demonstrated significant main effects of visual exposure for attention and abstract reasoning, whereas olfactory exposure demonstrated significant effects for memory recall and dishonest responding. Several outcomes also exhibited significant visual × olfactory interactions, suggesting that the effects of one sensory modality depended on the presence of the other. Prior research has suggested that sensory cues can influence decision-making processes through affective, associative, and contextual pathways (Hedblom et al., 2019; Huang and Kang, 2015; Monique et al., 2008; Bentley et al., 2023). One possible explanation is that nature-associated olfactory stimuli may evoke cognitive or emotional associations that influence behavioral choices. However, the present study did not directly assess moral cognition, implicit associations, or emotional mediation. Consequently, any mechanistic explanation for the observed reductions in dishonest behavior should be considered speculative. Future research incorporating direct measures of these processes would help clarify the pathways linking olfactory stimuli and behavioral decision-making. These effects are further supported by prior research on sensory processing in natural environments. Existing literature indicates that exposure to natural environments, particularly visual stimuli, can elicit positive affective responses, including increased engagement and reduced negative emotional indicators (Ekman, 1999). Similarly, olfactory stimuli have been shown to influence emotional processing through pathways associated with the limbic system (Ekman et al., 1987). Together, these findings support the interpretation that visual and olfactory stimuli may differentially influence cognitive and affective responses, reinforcing the distinct pathways observed in this study. The affective findings derived from AFFDEX facial-expression analysis should be interpreted cautiously. These measures were included as exploratory physiological indicators intended to provide a secondary context rather than serve as primary inferential outcomes. Accordingly, the facial-expression results are presented descriptively and are intended to complement, rather than independently substantiate, the behavioral findings observed across experimental conditions.

Second, although performance in the stress-inducing task did not differ significantly across conditions, physiological measures revealed consistent differences in blink rate. Participants in the nature exposure conditions generally exhibited lower blink rates than those in the control condition. In visually demanding tasks, lower blink rates have been associated with sustained visual attention and uninterrupted information processing, although blink-rate interpretation remains highly context dependent. The consistency of this pattern across nature exposure conditions suggests that visual and olfactory nature stimuli may have influenced attentional engagement during task performance, even in the absence of observable differences in behavioral outcomes.

### 8.1 Strengths and limitations

This study has several strengths. First, it employed a controlled experimental design with a relatively large sample size (*N* = 256), allowing for systematic comparison across four environmental conditions. Second, the use of incentivized behavioral tasks provides ecologically relevant measures of workplace-related functioning, including attention, memory, reasoning, risk preferences, and cheating behavior. Third, the integration of physiological measures offers complementary insight into underlying cognitive and affective processes beyond task-based outcomes. Importantly, this study extends existing literature by examining multisensory nature exposure, moving beyond the predominant focus on visual stimuli and demonstrating that olfactory cues may also influence behavioral outcomes such as dishonest decision-making. Additionally, supplementary mixed-effects models accounting for session-level clustering produced findings that were largely consistent with the primary factorial analyses, supporting the robustness of the observed effects. Finally, the inclusion of the short breaks between tasks enhances ecological validity. They mirror common workplace behavior, where employees may step away from their work environment to take a break. By varying the presence (or absence) of nature exposure during these breaks, the study more closely reflects how individuals engage with (or remain disconnected from) nature in real-world office settings.

On the other hand, several limitations must also be noted. First, participants were recruited from a university population, which may limit generalizability to broader workplace settings. Second, the olfactory stimulus was a commercially available fragrance designed to approximate natural environments. Consequently, observed effects may reflect responses to a pleasant nature-associated scent rather than exposure to authentic environmental olfactory stimuli. Third, physiological measures such as blink rate are inherently context-dependent and should be interpreted with caution, particularly given that additional indicators of ocular and psychological measures (e.g., blink duration) were not assessed. Finally, although the tasks were designed to reflect workplace-relevant behaviors, they remain laboratory-based measures and may not fully capture the complexity of real-world work environments. Future research should extend these findings to more real-world settings, incorporate additional physiological and behavioral measures, other senses of nature and further explore how multiple sensory modalities interact to influence cognitive and behavioral outcomes.

## 9 Conclusion

Collectively, these findings contribute to the growing literature on biophilic design by demonstrating that visual and olfactory nature stimuli are associated with improvements in specific cognitive and behavioral outcomes in workplace-relevant tasks through diverging pathways. Rather than producing uniform effects, the results highlight these distinct pathways through which different sensory modalities influence performance. Visual exposure demonstrated significant effects on attention and abstract reasoning, whereas olfactory exposure was associated with memory performance and dishonest responding. Several outcomes also exhibited significant interactions between visual and olfactory exposure, highlighting the importance of considering nature exposure as a multisensory process rather than evaluating individual sensory channels in isolation. Importantly, these findings suggest that relatively simple and low-cost environmental modifications can meaningfully shape cognitive functioning and decision-making processes in indoor work settings. Additionally, physiological indicators like reduced blink rates, observed increases in joy and engagement further suggest that incorporating nature into work may influence attentional engagement, perceived fatigue, focus and stress-related response. Taken together, this study portrays the value of multisensory approaches to biophilic design, suggesting that integrating both visual and olfactory elements (and perhaps other senses too) may enhance the effectiveness of workplace performance, and engagement, and overall productivity.

## Data Availability

The raw data supporting the conclusions of this article will be made available by the authors, without undue reservation.
