# Supplementary material for: An experimental study of the acute effects of visual and olfactory nature stimuli on task performance
Source: Front Psychol. 2026 Jul 13;17:1872507. doi: 10.3389/fpsyg.2026.1872507 (PMC13402151; doi:10.3389/fpsyg.2026.1872507)
Supplement: Supplementary file 1 [file Table_1.docx]

Supplementary Tables

# Supplementary Table 1: Emotions with corresponding action units and Facial Action Coding System (FACS) descriptions (1, 2)

| Emotion | Action Unit | FACS Description |
| --- | --- | --- |
| Joy | 6, 12 | Cheek raiser, lip corner pullers |
| Anger | 4,5,7,23 | Brow Lowerer, Upper lid raiser, lid tightener, lip tightener |
| Surprise | 1,2,5,26 | Inner brow raiser, outer brow raiser, upper lid raiser, jaw drop |
| Fear | 1,2,4,5,7,20,26 | Inner brow raiser, outer brow raiser, brow lowerer, upper lid raiser, lid tightener, lip stretcher, jaw drop |
| Sadness | 1,14,15 | Inner brow raiser, brow lowerer, lip corner depressor |
| Disgust | 9,15,16 | Nose wrinkler, lip corner depressor, lower lip depressor |
| Engagement | 1,2,4,6,9,12,15,17, 18,24,25,28 | Inner brow raiser, outer brow raiser, brow lowerer, Cheek raiser, Nose wrinkler, lip corner puller, lip corner depressor, chin raiser, lip puckerer, lip pressor, lips part, lip suck |

*Mapping of emotions to facial action units (AUs) based on the Facial Action Coding System (FACS). Each emotion is represented by a combination of facial muscle movements (action units), as defined by the FACS framework and implemented in the AFFDEX algorithm.*

# Supplementary Table 2: Demographic Distribution of sample population across conditions

| **Item** | **Freq** | **%** | **Freq** | **%** | **Freq** | **%** | **Freq** | **%** | **Χ^2^** | **p** |
| --- | --- | --- | --- | --- | --- | --- | --- | --- | --- | --- |
| **Condition** | **Control** | | **Views** | | **Scents** | | **Both** | |  |  |
| **SEX** | | | | | | | | | | |
| Male | 39 | 60.9 | 25 | 39.1 | 35 | 54.7 | 29 | 45.3 | 9.9 | 0.1 |
| Female | 25 | 39.1 | 39 | 59.3 | 29 | 45.31 | 35 | 54.7 |  |  |
| **AGE** | | | | | | | | | | |
| 18 – 23 | 48 | 75.0 | 51 | 79.7 | 53 | 82.8 | 55 | 85.9 | 5.7 | 0.5 |
| 24- 32 | 14 | 21.8 | 13 | 20.3 | 10 | 15.62 | 9 | 14.1 |  |  |
| 33 - 40 | 2 | 3.2 | 0 | -- | 1 | 1.56 | 0 |  |  |  |
| **RACE** | | | | | | | | | | |
| White | 12 | 18.7 | 12 | 18.7 | 16 | 25 | 19 | 29.6 | 15.2 | 0.6 |
| Black | 1 | 1.56 | 5 | 7.81 | 1 | 1.56 | 2 | 3.1 |  |  |
| Asian | 33 | 51.56 | 25 | 39.06 | 22 | 34.37 | 24 | 37.5 |  |  |
| Hispanics | 9 | 14.06 | 9 | 14.06 | 13 | 20.31 | 12 | 18.7 |  |  |
| Others | 9 | 12.5 | 12 | 18.75 | 10 | 15.62 | 7 | 10.93 |  |  |
| **PEAK PRODUCTIVITY TIME** | | | | | | | | | | |
| 8–10 AM | 10 | 15.63 | 21 | 32.8 | 14 | 21.87 | 9 | 14.06 | 16.6 | 0.1 |
| 11AM– 1PM | 28 | 43.75 | 31 | 48.43 | 31 | 48.4448.4 | 35 | 54.6954.6 |  |  |
| 3-5PM | 18 | 28.12 | 7 | 10.93 | 15 | 23.4423.4 | 10 | 15.63 |  |  |
| 7-9PM | 8 | 12.5 | 5 | 7.81 | 4 | 6.256.25 | 10 | 15.63 |  |  |
| **MORNING OR EVENING PERSON** | | | | | | | | | | |
| Definitely Morning | 10 | 15.63 | 12 | 18.75 | 13 | 20.31 | 10 | 15.63 | 8.4 | 0.5 |
| Rather a morning | 16 | 25.00 | 20 | 31.25 | 18 | 28.12 | 18 | 28.12 |  |  |
| Rather an evening | 26 | 40.62 | 14 | 21.87 | 22 | 34.38 | 26 | 40.62 |  |  |
| Definitely Evening | 12 | 18.75 | 18 | 28.13 | 11 | 17.19 | 10 | 15.63 |  |  |
| **STRESS LEVEL – PRE-STRESSOR** | | | | | | | | | | |
| Not at all Stressed | 28 | 43.75 | 36 | 56.25 | 31 | 48.44 | 32 | 50.0 | 7.0 | 0.6 |
| A little Stressed | 28 | 43.75 | 20 | 31.25 | 20 | 31.25 | 23 | 35.94 |  |  |
| Fairly Stressed | 8 | 12.50 | 7 | 10.94 | 10 | 15.63 | 7 | 10.94 |  |  |
| A lot Stressed | 0 | 0 | 1 | 1.56 | 3 | 4.68 | 2 | 3.12 |  |  |
| **STRESS LEVEL – POST STRESSOR** | | | | | | | | | | |
| No Change in Stress Level | 7 | 10.94 | 10 | 15.63 | 10 | 15.63 | 13 | 20.31 | 9.2 | 0.7 |
| A little less Stressed | 18 | 28.12 | 14 | 21.87 | 16 | 25.00 | 10 | 15.63 |  |  |
| A lot less Stressed | 9 | 14.06 | 5 | 7.81 | 5 | 7.81 | 6 | 9.37 |  |  |
| A little more Stressed | 23 | 35.94 | 21 | 32.81 | 24 | 37.50 | 22 | 34.38 |  |  |
| A lot more Stressed | 7 | 10.94 | 14 | 21.87 | 9 | 14.06 | 13 | 20.31 |  |  |
| **HOW DID MINI BREAKS AFFECT YOUR STRESS LEVELS? (Mental Reset)** | | | | | | | | | | |
| Didn’t affect | 17 | 26.56 | 26 | 40.62 | 23 | 35.94 | 26 | 40.62 | 11.9 | 0.2 |
| A little Change | 27 | 42.19 | 22 | 34.38 | 27 | 42.19 | 31 | 48.44 |  |  |
| Fair Amount | 18 | 28.12 | 13 | 20.31 | 13 | 20.31 | 5 | 7.81 |  |  |
| A lot | 2 | 3.13 | 3 | 4.69 | 1 | 1.56 | 2 | 3.13 |  |  |

*Table 2. Distributions of demographic variables across the four conditions. Chi-square tests indicate no statistically significant differences between groups, suggesting successful randomization.*

**Supplemental Table 3: One-way ANOVA Results across four conditions**

|  | | **Sum of Squares** | **df** | **Mean Square** | **F** |  | **Sig.** | **η²** |
| --- | --- | --- | --- | --- | --- | --- | --- | --- |
| **Task 1: Stress Test / Mental Math** | Between Groups | .074 | 3 | .025 | .42 |  | .734 |  |
|  | Within Groups | 14.6 | 252 | .058 |  |  |  | 0.005 |
|  | Total | 14.6 | 255 |  |  |  |  |  |
| **Task 2: Attention Retention / Letter ID** | Between Groups | 4.6 | 3 | 1.5 | 5.89 |  | <.001 | 0.066 |
|  | Within Groups | 66.0 | 252 | .26 |  |  |  |  |
|  | Total | 70.7 | 255 |  |  |  |  |  |
| **Task 3: Memory Recall / Wordlist Learning** | Between Groups | 40.76 | 3 | 13.58 | 12.28 |  | <.001 | 0.128 |
|  | Within Groups | 278.71 | 252 | 1.106 |  |  |  |  |
|  | Total | 319.48 | 255 |  |  |  |  |  |
| **Task 4: Abstract Reasoning / Raven’s Matrices** | Between Groups | 15.3 | 3 | 5.1 | 2.86 |  | .038 | 0.033 |
|  | Within Groups | 451.6 | 252 | 1.7 |  |  |  |  |
|  | Total | 467 | 255 |  |  |  |  |  |
| **Task 5: Precautionary Risk** | Between Groups | .480 | 3 | .160 | 2.62 |  | .051 |  |
|  | Within Groups | 15.3 | 252 | .061 |  |  |  | 0.030 |
|  | Total | 15.8 | 255 |  |  |  |  |  |
| **Task 6: Honesty** | Between Groups | 32.1 | 3 | 10.7 | 5.74 |  | <.001 | 0.064 |
|  | Within Groups | 468.1 | 251 | 1.8 |  |  |  |  |
|  | Total | 500.3 | 254 |  |  |  |  |  |

*One way ANOVA. Between-group differences in task performance across the four conditions. Significant effects (p < 0.05) indicate differences in mean performance across conditions. Effect sizes are reported as η².*

# Supplementary Table 4: Tukey HSD pairwise comparisons

| **Tukey’s HSD** | | | | | | | |
| --- | --- | --- | --- | --- | --- | --- | --- |
| **Dependent Variable** | **Condition case (I)** | **Condition case (II)** | **Mean Difference (I-II)** | **Std. Err** | **Sig.** | **95% Confidence Interval** | |
|  |  |  |  |  |  | Lower Bound | Upper Bound |
| Task 2: Attention | Views | Control | 0.375* | 0.091 | <0.001 | 0.14 | 0.61 |
|  |  | Scents | 0.234* | 0.091 | 0.050 | 0.00 | 0.47 |
| Task 3: memory | Control | Views | -0.875* | 0.185 | <0.001 | -2.67 | -1.74 |
|  |  | Scents | -1.047* | 0.185 | <0.001 | -2.32 | -1.55 |
|  |  | Both | -0.547* | 0.185 | <0.001 | 2.08 | 2.67 |
|  | Views | Scents | -0.171 | 0.185 | .792 | .05 | .64 |
|  |  | Both | 0.328 | 0.185 | 0.293 | .24 | .82 |
|  | Scents | Both | 0.501* | 0.185 | <0.05 | 0.192 | 0.980 |
| Task 4: Reasoning | Control | Both | -.688^*^ | .237 | .021 | -1.30 | -.08 |
| Task 6: Honesty | Control | Scents | .984^*^ | .241 | <.001 | .36 | 1.61 |
| *: The mean difference is significant at the 0.05 level. | | | | | | | |

*Pairwise comparisons between conditions for tasks with significant ANOVA results. Values represent mean differences (I–II), standard errors, p-values, and 95% confidence intervals. Only statistically significant comparisons are shown.*

# Supplementary Table 5: Global presence (%) of negative emotional responses across experimental conditions.

| Emotions/Group​ | Disgust​ | Contempt​ | Fear​ | Anger​ | Confusion​ | Sadness​ |
| --- | --- | --- | --- | --- | --- | --- |
| Control​ | 0.22​ | 2.31​ | 0.99​ | 3.35​ | 0.69​ | 0.58​ |
| Views​ | 1.09​ | 0.30​ | 0.05​ | 2.19​ | 1.64​ | 0.67​ |
| Scents​ | 1.13​ | 1.82​ | 0.98​ | 5.37​ | 2.74​ | 3.64​ |
| Both​ | 0.56​ | 1.24​ | 3.9​ | 3.042​ | 1.88​ | 1.95​ |

*Values represent the percentage presence of negative emotional responses derived from AFFDEX analysis across all tasks. No statistically significant differences were observed between conditions.*

**Supplemental Table 6: Mixed-effects models accounting for session-level clustering.**

| Outcome | ICC | Views p | Scent p | Interaction p |
| --- | --- | --- | --- | --- |
| Stress | 0.073 | .444 | .491 | .758 |
| Attention | 0.013 | .003 | .820 | .027 |
| Memory | 0.023 | .189 | .017 | <.001 |
| Reasoning | 0.029 | .082 | .065 | .664 |
| Risk | 0.062 | .848 | .883 | .020 |
| Cheating | 0.003 | .969 | .002 | .030 |

# References

1. Farnsworth B. How to measure emotions and feelings (and the difference between them). Imotions. <https://imotions>. com/blog/difference-feelings-emotions; 2020.

2. Kjærstad HL, Jørgensen CK, Broch-Due I, Kessing LV, Miskowiak K. Eye gaze and facial displays of emotion during emotional film clips in remitted patients with bipolar disorder. Eur Psychiatry. 2020;63(1):e29.
